# Medicaid Expansion and In-Hospital Cardiovascular Mortality Failure or Unrealistic Expectations?

**DOI:** 10.1001/jamanetworkopen.2018.1303

**Published:** 2018-08-03

**Authors:** Rishi K. Wadhera, Karen E. Joynt Maddox

**Affiliations:** Brigham and Women’s Hospital Heart & Vascular Center, Harvard Medical School, Boston, Massachusetts (Wadhera); Richard and Susan Smith Center for Outcomes Research in Cardiology, Division of Cardiology, Beth Israel Deaconess Medical and Harvard Medical School, Boston, Massachusetts (Wadhera); Cardiovascular Division, Department of Medicine, Washington University School of Medicine in St Louis, St Louis, Missouri (Joynt Maddox).

A central objective of the Affordable Care Act was to expand insurance coverage for low-income, uninsured Americans. Prior to the Affordable Care Act, eligibility for Medicaid varied greatly among states. Texas, for example, did not offer Medicaid insurance to poor adults without children, but the same adults living in Maine were eligible for Medicaid if they earned less than 100% of the federal poverty level in income. To ensure that low-income persons could access insurance regardless of their state of residence, the Affordable Care Act required states to expand Medicaid eligibility to any individual with an income at or below 138% of the federal poverty level. A subsequent US Supreme Court ruling in 2012, however, effectively made Medicaid expansion optional rather than mandatory. To date, 33 states (including the District of Columbia) have expanded Medicaid, while 18 states have elected to not implement expansion. This distinction has created an opportunity for researchers and policy makers to evaluate the relationship between Medicaid expansion and care delivery and outcomes.

Akhabue and colleagues^1^ do just that, exploring whether uninsured hospitalizations for major cardiovascular events (acute myocardial infarction, heart failure, and stroke) and in-hospital mortality changed after states implemented Medicaid expansion, compared with states that elected to not expand Medicaid. To do so, they evaluated rates of uninsured and Medicaid hospitalizations among all non-Medicare hospitalizations from 17 expansion states and 13 nonexpansion states in the years preceding expansion (2009–2013) and the year after expansion (2014).

Overall, the authors found that among expansion states, the proportion of uninsured hospitalizations declined by 5.0% (95% CI, −6.2% to −3.8%) and Medicaid hospitalizations increased by 10.2% (95% CI, 8.8% to 11.6%). In contrast, among nonexpansion states, the proportions of uninsured and Medicaid hospitalizations were unchanged. A multivariable adjusted difference-indifferences analysis demonstrated that expansion states experienced a significant reduction in uninsured hospitalizations after expansion relative to nonexpansion states (adjusted difference-indifferences estimate, −5.8%; 95% CI, −7.5% to −4.2%; *P* < .001), as well as a significant increase in Medicaid hospitalizations (adjusted difference-in-differences estimate, 8.4%; 95% CI, 6.5% to10.2%; *P* < .001).

There are a number of reasons one might think insurance coverage would translate into better in-hospital outcomes. First, uninsured patients lack longitudinal, reliable access to outpatient care services required to identify and treat comorbid conditions. Insurance could plausibly improve outpatient management and reduce severity at presentation, reducing in-hospital mortality. In addition, lack of insurance is associated with delays in seeking emergency care in part out of concern for financial liability; insurance could remove this important barrier. Finally, prior studies have shown that uninsured patients hospitalized for acute cardiovascular conditions are less likely to receive guideline-directed medical therapy, aggressive care, and invasive cardiac procedures, which may explain their worse outcomes compared with insured patients.^2,3^ Insurance could influence the care delivered during hospitalization by removing any financial barriers to optimal care delivery.

However, Akhabue and colleagues observed no significant change in in-hospital mortality in expansion vs nonexpansion states during the study period. Does this mean that Medicaid expansion was a failure? Have we put billions of dollars into health reform for no good reason?

To the contrary, a growing body of evidence suggests that Medicaid expansion has had a number of benefits. Medicaid expansion is associated with greater access to primary and preventive care in the outpatient setting, as well as better detection and treatment of some chronic conditions such as depression.^4,5^ This perhaps explains why expansion has also been associated with improved self-reported health.^4,6^ For cardiovascular care in particular, the identification and treatment of risk factors, such as high cholesterol level, hypertension, and diabetes, have improved since expansion,^7,8^ as has the use of prescription cardiovascular drugs.^4,9^ It is possible that the single postexpansion year examined by Akhabue and colleagues was too short to appreciate the incremental, cumulative health benefits of access to preventive care, medications, and treatment of chronic illnesses; it is also possible that better care of chronic illness before an acute exacerbation might not be associated with better outcomes for that event. Or, perhaps in the immediate aftermath of insurance expansion, we are seeing pent-up demand, and longer follow-up will be required before these patterns settle out. Future research should evaluate more years after expansion, all states, and outcomes in the period following discharge to provide a comprehensive picture of expansion and outcomes in the context of acute hospitalization.

Finally, the most important aspect of Medicaid expansion may not be a direct health effect at all. Insurance protects against unbearable financial risk associated with health care costs, particularly among low-income individuals who are most susceptible, which explains why Medicaid expansion has been associated with reduced catastrophic expenditures, out-of-pocket spending, and bankruptcies.^5^ In this context, the decline in uninsured cardiovascular hospitalizations observed by Akhabue and colleagues in expansion states is extremely important regardless of whether it changed inpatient outcomes, as prior to Medicaid expansion, an estimated three-quarters of uninsured persons hospitalized for acute myocardial infarction or stroke in the United States experienced catastrophic health expenditures.^10^ Protection against bankruptcy brought on by cardiovascular disease may have many positive downstream effects, both for individuals and for society more broadly.

Akhabue and colleagues′ investigation comes at a time when Medicaid expansion is particularly contentious. Recently, residents of Maine decisively voted to expand Medicaid even further, although the state’s governor is now being sued for refusing to do so. Other states, such as Idaho, are considering moving forward with efforts to expand. As such, Akhabue and colleagues make an important contribution to our understanding of Medicaid expansion and acute hospitalizations for cardiovascular conditions at a time when it is vital that evidence inform the ongoing policy debate.
